# Teleneurology expertise in intensive care units across Germany - a nationwide survey

**DOI:** 10.1186/s42466-025-00451-7

**Published:** 2025-11-24

**Authors:** Eyad Altarsha, Kristian Barlinn, Albrecht Günther, Hans Worthmann, Karl-Georg Häusler, Christian Urbanek, Benjamin Büchele, Torsten Kraya, Stefan Merkelbach, Mazen Abu-Mugheisib, Bernd Kallmünzer, Philipp Zickler, Florian Schöberl, Jürgen Bardutzky, Julian Bösel, Heinrich J. Audebert, Gordian J. Hubert, Hagen B. Huttner, Christoph Gumbinger, Jessica Barlinn

**Affiliations:** 1 Department of Neurology, Faculty of Medicine, University Hospital Carl Gustav Carus, Technische Universität Dresden, Fetscherstrasse 74, 01307 Dresden, Germany; 2 Department of Neurology, University Hospital Jena, Jena, Germany; 3 Department of Neurology, Hannover Medical School, Hannover, Germany; 4 Department of Neurology, University Hospital Ulm, Ulm, Germany; 5 Department of Neurology, Ludwigshafen Municipal Hospital, Ludwigshafen, Germany; 6 Department of Neurology, Karlsruhe Municipal Hospital, Karlsruhe, Germany; 7 Department of Neurology, St. Georg Hospital Leipzig, Leipzig, Germany; 8 Department of Neurology, University Hospital Halle Saale, Halle, Germany; 9 Department of Neurology, Heinrich Braun Hospital Zwickau, Zwickau, Germany; 10 Department of Neurology, Braunschweig Municipal Hospital, Braunschweig, Germany; 11 Department of Neurology, University Hospital Erlangen, Erlangen, Germany; 12 Department of Neurology and Clinical Neurophysiology, University Hospital Augsburg, Augsburg, Germany; 13 Department of Neurology, LMU University Hospital Munich-Großhadern, München, Germany; 14 Department of Neurology, University Hospital Freiburg, Freiburg, Germany; 15 Department of Neurology, Friedrich Ebert Hospital Neumünster, Neumünster, Germany; 16 Department of Neurology, University Hospital Heidelberg, Heidelberg, Germany; 17 Faculty of Neurology, Neurocritical Care Division, Johns Hopkins University Hospital, Baltimore, MD USA; 18 Department of Neurology and Experimental Neurology, Charité - Universitätsmedizin Berlin, Berlin, Germany; 19 Center for Stroke Research Berlin, Charité - Universitätsmedizin Berlin, Berlin, Germany; 20 Department of Neurology, München-Klinik Harlaching, München, Germany

**Keywords:** Telemedicine, Neurocritical care, Teleneurology, Stroke networks

## Abstract

**Background:**

Telemedicine is well established in acute stroke care and significantly contributes to widespread access to treatment. In intensive care, telemedicine is increasingly used to reduce mortality and complications. The German Society of Anesthesiology and Intensive Care Medicine (DGAI) also recommends telemedical consultations for neurological indications.

**Methods:**

The aim of this survey was to assess structure, usage and need for telemedicine consultations for non-neurologically managed intensive care units and to determine whether there is a need to expand telemedicine stroke networks to include neurointensive care. A national cross-sectional survey was conducted, targeting all 22 German telemedicine stroke networks. The survey included 27 questions on structural aspects of intensive care units, the utilization of telemedical consultations and experiences with tele-neurointensive diagnostics and therapy. Additionally, a sub-study was conducted in six spoke hospitals within the telemedicine stroke network East Saxony (SOS-TeleNET).

**Results:**

Of the 22 networks contacted, 17 (77%) responded. Of these, 11 (65%) regularly received consultation requests from intensive care units, most of which were handled by teleneurologists. The most common indications consisted of ischemic and hemorrhagic strokes, epileptic seizures as well as prognosis assessment and therapy goal adjustments. Several networks indicated interest in expanding telemedicine services for neurological care in intensive care units.

**Conclusions:**

The survey highlights a notable need for telemedicine neurointensive care consultations. Expanding telemedicine infrastructure in this field could contribute to improving the quality of care.

**Supplementary Information:**

The online version contains supplementary material available at 10.1186/s42466-025-00451-7.

## Introduction

Since the early 2000s, telemedicine has become an essential element of stroke care in Germany and now plays a major role in providing nearly nationwide access to specialized services [1]. Currently, around one in ten stroke patients is treated with telemedical support, as shown in a recent survey of the national telemedicine stroke networks [2]. Hospitals participating in these networks achieve thrombolysis rates and referral frequencies for thrombectomy that are comparable to those of neurologist-led stroke units [2].

In intensive care medicine, ensuring high-quality care close to home remains a key challenge, especially given the growing demand for specialized personnel and subspecialties. Neurocritical care, in particular, is limited to a few centers across Germany. Telemedical approaches have already been shown to reduce mortality, complication rates and intensive care unit (ICU) length of stay [3, 4]. The current guideline of the German Society for Anaesthesiology and Intensive Care Medicine (DGAI) recommends the use of teleconsultations for neurological conditions such as stroke, intracerebral hemorrhage, status epilepticus and traumatic brain injury [5].

Neurocritical care involves the management of life-threatening neurological conditions such as severe ischemic or hemorrhagic stroke, status epilepticus, meningoencephalitis or neuromuscular crises. As dedicated neurocritical care units remain scarce in Germany, most neurologically ill ICU patients are treated in general interdisciplinary units. In addition, there is a substantial shortage of trained neurointensivists [6]. In this context, telemedical support for non-neurologist-led ICUs could be a valuable addition to guideline-based care for neurologically ill patients. Neurological complications such as delirium, prolonged weaning or critical illness polyneuropathy/myopathy including dysphagia, can significantly affect ICU outcomes.

Outside Europe, teleneurocritical care has been increasingly integrated into ICU pathways, particularly in the United States, where programs provide round-the-clock expert input for stroke, seizures and broader neurocritical indications. Observational studies report functional outcomes comparable to bedside neurocritical care in selected ICU populations and economic analyses show equivalent billable charges to in-person care [7–9].

However, it is currently unclear whether there is a demand for neurocritical care consultations or whether relevant structures - such as extensions of existing telemedicine stroke networks - already exist. To assess the current situation and evaluate the need for teleneurocritical care, we conducted a survey study within the German telemedicine stroke networks.

## Methods

**Fig. 1:**
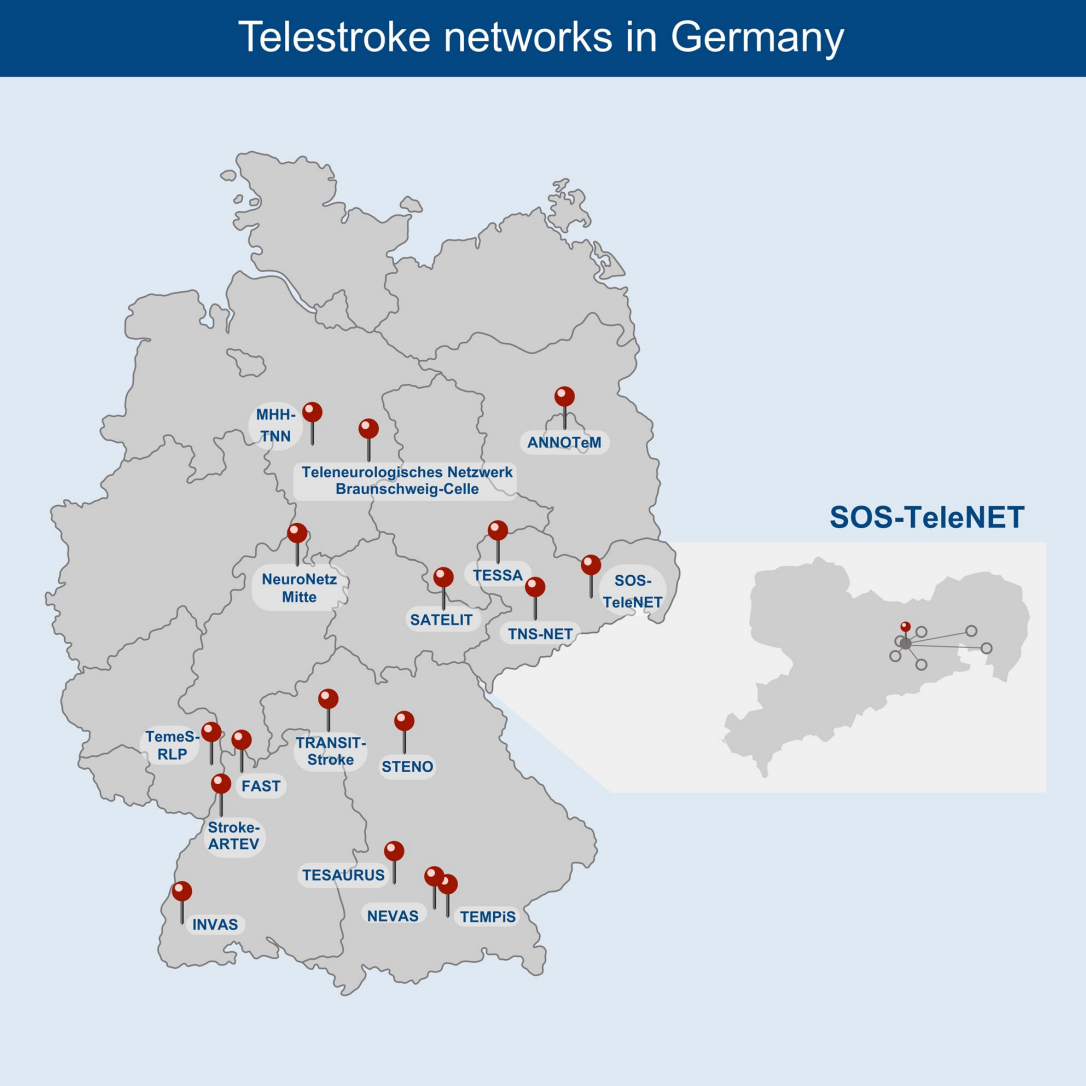
Stroke networks participating in the survey and cooperating hospitals participating in the sub-study within the SOS-TeleNet stroke network

A national cross-sectional survey was conducted, targeting all 22 telemedicine stroke networks in Germany (Fig. 1). These networks are organized under the Commission for Telemedicine Stroke Care of the German Stroke Society [10]. A concise description of these networks is available in a recent national survey and in Supplementary Table S1 [2]. The aim of the study was to assess the current structures, usage and demand for teleneurocritical consultations for ICU patients.

A questionnaire was specifically developed for this study, comprising a total of 27 multiple-choice and hybrid questions (including single-answer, semi-open and open-ended questions) to capture both quantitative and qualitative aspects of tele-intensive care. Content validity was ensured through expert review. A pretest was conducted in three network centers to assess clarity and technical functionality. Based on the feedback, minor adjustments were made to improve the clarity and unambiguity of the questions. The questionnaire captured typical practice patterns and frequency categories rather than counts over a predefined time window. The full questionnaire is provided in the Supplement (Survey S1/S2).

Data collection took place between September 1 and November 30, 2021. The questionnaire was created using the online survey tool SurveyMonkey^®^ (San Mateo, USA) and distributed via individualized email links to the respective network coordinators. To improve the response rate, a structured follow-up process was implemented. This included an email reminder four weeks after the initial invitation, followed by targeted telephone calls in cases of non-response after an additional four weeks.

The questionnaire covered the following main topics:

- Structural characteristics of the network centers, particularly regarding neurocritical care.
- Use and implementation of teleneurocritical consultations for ICU patients.
- Experience with teleconsultation in diagnostic and therapeutic decision-making.
- Potential future applications of teleconsultation services.
- Training and continuing education offerings in neurocritical care.

In parallel with the nationwide survey, a substudy was conducted to assess the demand for teleneurocritical consultations at cooperating hospitals within one telestroke network. For this purpose, six cooperating hospitals of the Eastern Saxony Telemedicine Stroke Network (SOS-TeleNet) were included in a separate survey (Fig. 1). A dedicated questionnaire was developed for this substudy, consisting of nine multiple-choice and hybrid questions (single-answer, semi-open, and open-ended formats). The main topics addressed were: the structural capacity for intensive care at the participating hospitals, the perceived need for teleneurocritical consultations and the need for training in neurocritical nursing care. The full questionnaire is also provided in the online supplementary material.

### Statistical analysis

Data analysis was performed descriptively using STATA (Version 12.1, StataCorp, College Station, TX, USA). For continuous variables, the median and interquartile range (IQR) were calculated. Categorical variables were analyzed using absolute and relative frequencies. Missing values within each topic section were excluded from the analysis. All datasets were checked for completeness and plausibility prior to analysis.

## Results

### Response rate and network participation

Of the 22 telemedicine stroke networks contacted across Germany, 17 (77.3%) completed the online questionnaire. Among these, 10 out of 17 (58.8%) participating centers were university hospitals. All participating centers provided complete responses to each questionnaire item.

### Neurocritical care infrastructure in participating networks

Twelve of the surveyed centers (70.6%) reported operating a dedicated neurological intensive care unit (NICU). The average NICU bed capacity was 10 beds (IQR: 8.5–12). Eight centers (66.7%) had 5 to 10 beds, while four (33.3%) provided at least 12 beds. One center reported operating a 16-bed NICU. Not all NICU beds were equipped for mechanical ventilation. In total, the surveyed centers provided 119 NICU beds, of which 112 (94.1%) were ventilator-capable. The average number of ventilator-capable NICU beds was 10 (IQR: 8–11).

In the five centers without a dedicated NICU, neurological ICU patients were treated in anesthesiology and/or internal medicine intensive care units. These units had appropriate capacity for the care of neurologically ill patients. Four of these centers (80%) had more than 20 ventilator-capable ICU beds, while one (20%) had fewer than 20 beds. Neurological care was primarily provided by neurologists. Four of the five centers reported having at least one board-certified neurologist with an additional board certification in neurocritical care.

### Demand and practice of teleneurocritical care consultations

Eleven of 17 networks (64.7%) reported receiving neurocritical care consultation requests from affiliated hospitals. Among these 11 networks, 11 (100%) provided consultations on an on-demand, 24/7 basis without fixed scheduled rounds, whereas 1 (9.1%) additionally offered scheduled teleconsultation sessions (e.g., once per week). Four of 11 (36.4%) networks provided annual volume estimates: 1/4 reported > 40 teleneurocritical care consultations per month, while 3/4 reported 5 to 10 per month. Regarding provider qualifications, 6/11 (54.5%) indicated that consultations were handled by teleneurologists who are also the primary consultants for acute stroke within the networks; 4/11 (36.4%) reported provision by attending or senior physicians with training in neurocritical care; and 1/11 (9.1%) by residents. Only 3/11 (27.3%) reported that consultations were provided by neurologists with board certification in neurocritical care.

In four centers (36.4%), consultations were conducted exclusively via telephone. Six centers (54.5%) also used audio-video communication for teleconsultations. One center reported that consultations were performed exclusively through in-person assessments, either by in-house neurologists or in collaboration with local neurologists. Reported coverage was 24/7 and did not systematically vary by day/night or weekday/weekend. In centers performing teleconsultations, the telemedicine equipment is typically relocated from the emergency department to the ICU, as dedicated telemedicine infrastructure is often lacking in ICU settings. All six centers performing full teleconsultations were able to assess brain imaging (CT/MRI) after transmission. None of the surveyed networks reported offering remote EEG interpretation.

**Fig. 2:**
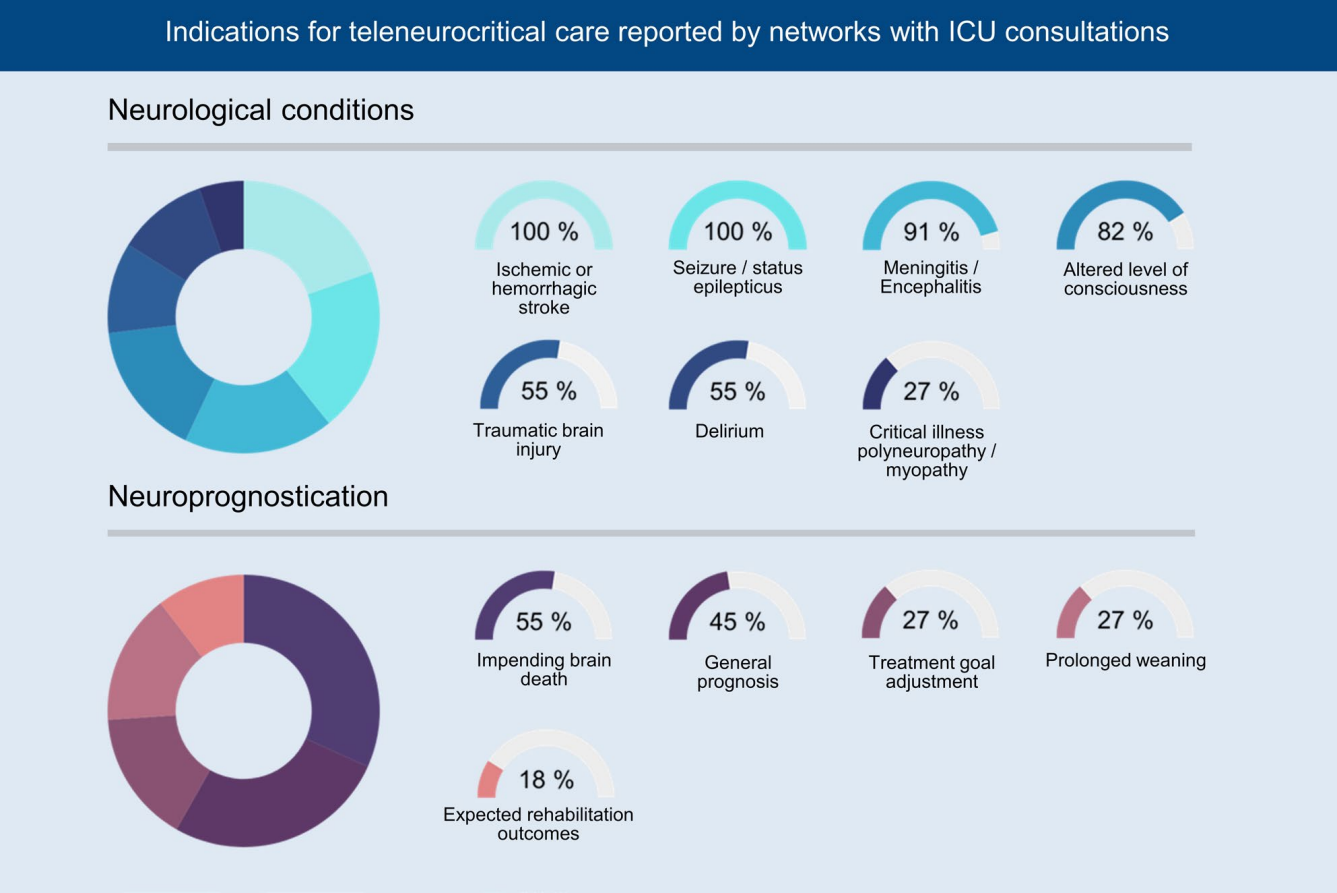
Values show the percentage of networks (denominator = 11) that reported receiving teleneurocritical care consultations for each indication. Multiple responses were allowed; therefore, percentages do not sum to 100. Call-level counts by indication were not collected

**Fig. 3:**
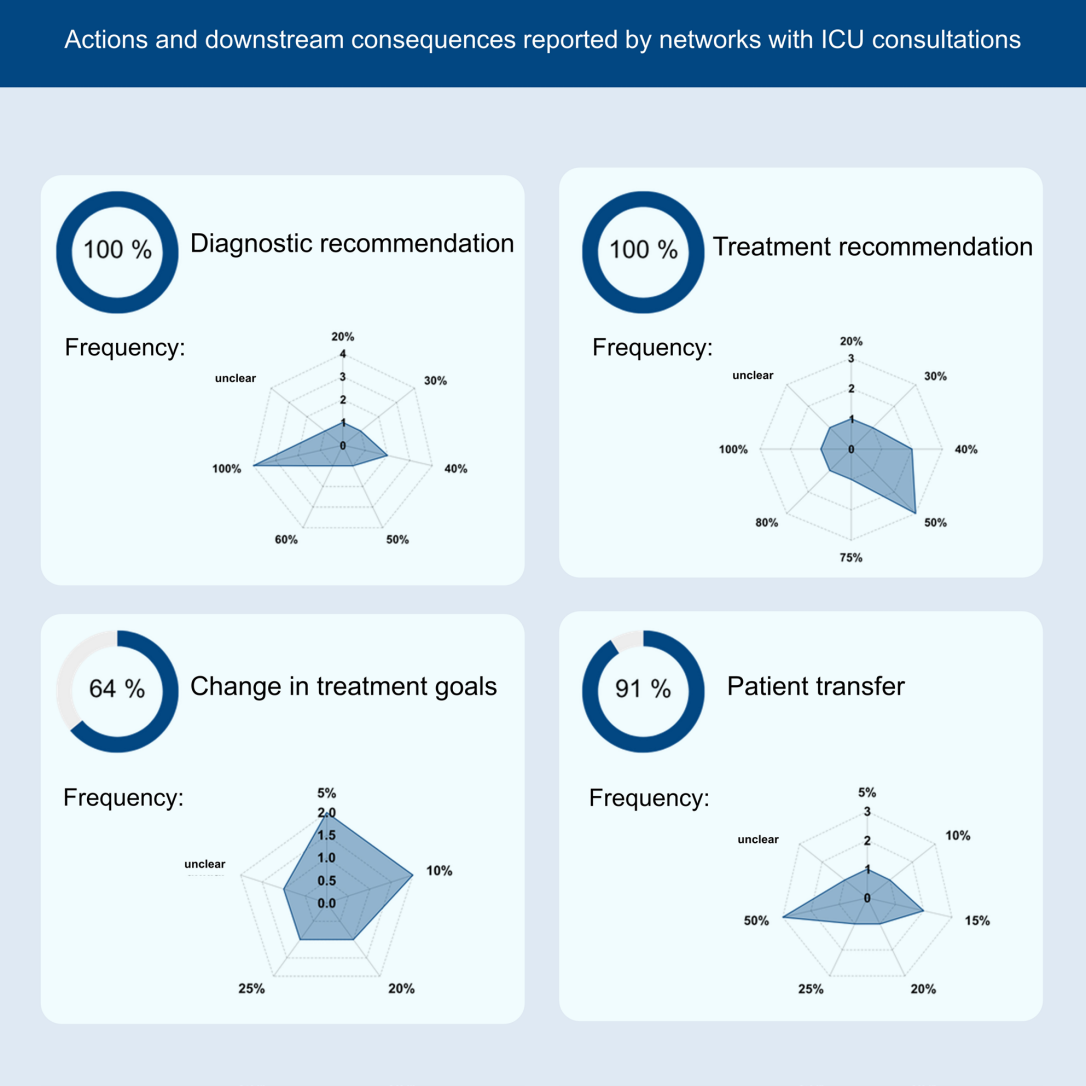
Values show the percentage of networks (overall denominator = 11) that reported each action/outcome (e.g., diagnostic or treatment recommendations; patient transfer occurring in some consultations). These are network-level indicators. For each panel, the badge displays the number of reporting networks (e.g., 7/11). The radar (“spider web”) plot shows the distribution of frequency categories among those reporting networks only and the radius encodes the count of networks in that category

All 11 networks (100%) indicated that most consultations addressed conditions already covered by the stroke network, especially ischemic and hemorrhagic strokes. Most also reported receiving consultations on other neurological conditions and neuroprognostication (Fig. 2). All centers regularly provided diagnostic and treatment recommendations during consultations. In addition, most centers reported offering guidance on goals-of-care discussions, therapy limitation and patient transfers. The frequency of these recommendations varied between centers. Figure 3 shows the distribution of topics addressed in neurocritical consultations.

Of the six networks that did not regularly receive ICU consultation requests from affiliated hospitals, four (66.7%) expressed interest in establishing a tele-neurocritical consultation service. These centers identified stroke (ischemic and hemorrhagic), treatment limitation and prognosis, disorders of consciousness and seizures/status epilepticus as particularly relevant indications (100%). Prolonged weaning and rehabilitation potential were considered relevant by three centers each (75%). Delirium was identified by two centers (50%) and traumatic brain injury as well as CIP/CIM by one center each (25%). Three centers (75%) indicated interest in teleconsultations for guidance on the possible or anticipated diagnosis of brain death. Three of the four centers (75%) preferred to provide consultations for acute cases on demand, while standard coverage should be limited to regular working hours.

### Consultations on impending brain death

Six of the 11 networks (54.6%) reported receiving regular consultation requests related to the diagnosis or management of potentially impending brain death. In two centers, telephone consultation with imaging review was provided; one of these also offered audio-video teleconsultation. In the remaining four centers (66.7%), consultations were performed in person by neurologists from the network visiting the cooperating hospitals. One center (16%) had a designated brain death team. None of the centers initiating brain death consultations reported contacting the local transplant coordinators. Three centers (50%) stated that they would involve the German organ procurement organization (DSO) after initial consultation if necessary for further evaluation and coordination.

### Reimbursement of neurocritical consultations

Only two of the surveyed networks (18.2%) reported receiving separate reimbursement for neurocritical care consultations. This was achieved through existing framework agreements (e.g., for unclear neurological conditions) or through individual contracts with affiliated hospitals covering ICU-related neurological consultations. The low share of separate reimbursement streams refers to non-stroke teleneurocritical care; by contrast, stroke-associated teleconsultations are typically reimbursed under existing telestroke arrangements (spoke-side OPS billing and regional contracts) [2].

### Education and nursing support in neurocritical care

Of the 17 networks, 13 (76.5%) expressed an interest in involving ICU nursing staff in training or consultation programs. Key areas identified included neurological monitoring and scoring (100%), dysphagia management in neurological patients (92.3%) and analgesia, sedation, and delirium management (92.3%).

### Substudy on the demand for teleneurocritical consultations in spoke telestroke hospitals

All six hospitals participating in the substudy had anesthesiology-led ICUs; one also operated an internal medicine ICU. The average number of ICU beds was 9.5 (IQR: 8–13), with an average of 9.5 ventilator-capable beds (IQR: 6–10).

Half of the hospitals (50%) had an in-house neurological consultation service, while 33.3% relied on external providers and 16.7% on outpatient neurologists. Consultations were requested on demand, primarily for disorders of consciousness (100%), epilepsy (100%), stroke (83.3%), traumatic brain injury (50%), delirium (50%), and prolonged weaning (33.3%). Common neuroprognostic topics included irreversible brain function loss (83.3%), general prognosis estimation (83.3%), rehabilitation potential (83.3%), and treatment limitation (33.3%).

Spoke hospitals identified stroke (66.7%) and suspected or impending brain death (66.7%) as the most pressing indications for teleneurocritical consultations, followed by prognosis estimation (50%) and therapy limitation (50%). They self-estimated a median of 1.75 teleneurocritical care consultations per month (IQR: 1.5–4).

From a nursing perspective, the most relevant topics for consultation or training were neurological monitoring and scoring (66.7%), dysphagia management (66.7%), and analgesia, sedation, and delirium management (50%).

## Discussion

The results of this survey conducted in Germany indicate that two-thirds of established stroke networks already receive consultation requests from intensive care units of affiliated partner hospitals. These are typically addressed via telephone and in individual cases, through on-site visits. Only six centers currently provide neurocritical care consultations via telemedicine using audio-video communication, which also enables remote imaging review. Financial reimbursement for these services is secured in only two of the networks.

Within established stroke networks, affiliated hospitals have low-threshold access to neurocritical care expertise, as they can draw on existing infrastructure and well-established communication channels. For more than two decades, telemedicine-based stroke networks have contributed to ensuring acute stroke care in neurologically underserved, often rural areas [2]. In contrast, there is no structured telemedical care for neurocritical care patients in Germany. However, our findings indicate a clear demand for such a structure. This need, as expressed by the coordinating centers, must be interpreted in the context of the generally limited availability of neurocritical care expertise [6]. Neurologically managed ICUs remain rare and although the formal qualification in neurocritical care can be obtained through medical boards, it is not broadly integrated into clinical training.

It is important to distinguish teleneurocritical care from telestroke and general teleneurology. Telestroke primarily addresses hyperacute cerebrovascular pathways (reperfusion decisions and transfer coordination) within narrow time windows. By contrast, teleneurocritical care targets the broader, ongoing management of neurologically ill ICU patients - e.g., refractory status epilepticus, neuroprognostication (including suspected brain death), secondary neurological complications (delirium, critical-illness polyneuropathy, dysphagia) and goal-of-care discussions - often beyond the initial triage phase [7–9]. Compared with ad-hoc teleneurology, teleneurocritical care requires continuous availability, integration with ICU workflows (ventilation, sedation, hemodynamics) and close collaboration with critical-care teams. Pros of teleneurocritical care include timely specialist input beyond reperfusion care, standardized protocols and the potential to avoid transfers when appropriate; limitations include heterogeneous reimbursement, staffing demands and implementation variability.

Telemedical evaluation of critically ill neurological patients, including audiovisual assessments and imaging review, can be implemented relatively easily within the existing infrastructure of stroke networks. The required technical framework is already in place for acute stroke care and could be adapted for neurocritical care. EEG transmission is currently not part of routine telemedical care, although viable technical solutions exist and could be implemented.

Telemedicine has proven to be a robust and reliable means of delivering specialist expertise, supporting the provision of high-quality care across broad geographic areas. Studies conducted during the COVID-19 pandemic demonstrate that telemedical stroke care remained accessible and functional throughout the crisis [11, 12]. Beyond pandemic-specific contexts, high levels of acceptance for tele-ICU consultation services have also been observed in general ICU populations [3]. Potential benefits of tele-neurointensive consultations include reductions in hospital mortality and ICU length of stay. In a cohort of more than 6,000 patients from various ICUs, significant improvements were seen in key quality indicators such as complication rates, mortality and ICU stay duration [3]. A subsequent controlled multicenter study including over 100,000 patients and data from neurological ICUs confirmed these findings [13]. Early involvement of specialists, development of structured treatment plans, adherence to evidence-based guidelines, and interdisciplinary collaboration emerged as key factors in improving outcomes. These observations are consistent with evidence that care in dedicated neurocritical care units is associated with lower mortality compared with general ICUs, and that teleneurocritical care can achieve outcomes comparable to in-person neurocritical care in selected ICU stroke pathways [7–9, 14].

The ERIC (Enhanced Recovery after Intensive Care) study demonstrated the effectiveness of telemedical support in improving the quality of ICU care [16, 17]. Significant improvements were observed in seven out of eight quality indicators defined by the German Interdisciplinary Association for Intensive and Emergency Medicine (DIVI), with the strongest effect seen in the indicator “early weaning from mechanical ventilation.” These effects are likely transferable to neurocritical care settings. The findings from ERIC contributed to the development of a new policy on intensive care centers, issued by the Federal Joint Committee (G-BA) in October 2023 [17]. These centers are intended to strengthen interdisciplinary and interprofessional care, as well as the regional coordination of intensive care services. Tele-intensive care is a core element of these networks and the inclusion of neurologically led ICUs and neurological expertise is seen as particularly important.

Neuroprognostication also represents a central component of neurocritical consultations, particularly in cases of primary or secondary brain injury. The latter are often managed on non-neurological ICUs, where prognosis regarding long-term functional outcome frequently requires specialized neurological expertise [6]. Telemedicine could provide accessible neurointensive expertise to guide diagnostic and therapeutic pathways, especially in the context of prognostically driven decisions such as goal-of-care transitions [18].

In this context, early recognition of potentially impending brain death is also relevant. Over 80% of surveyed centers reported impending brain death as an indication for neurointensive consultation. A persistent deficit in detection of patients who potentially progress toward brain death significantly contributes to the ongoing low organ donation rate in Germany [19]. Initial studies have examined the use of telemedicine to support brain death diagnostics [20, 21]. Implementation of bidirectional audio-video teleconsultation in ICUs was associated with an increase in the identification of potential organ donors. One study reported that after the introduction of a teleneurology service, the proportion of patients fulfilling brain death criteria and medically eligible for organ donation increased 2.6-fold (8.9% vs. 21.1%) and realized postmortem donations increased 2.1-fold (6.1% vs. 13.1%) [21]. Brain death determination is time-critical and timely expert evaluation may increase the likelihood of family consent and successful organ donation [22]. The median time between teleconsultation request and initiation of consultation in the cited study was 20.2 min, underlining the rapid availability of neurological expertise. Notably, telemedicine was used not only for prognostic assessments but also for direct supervision of the brain death diagnostic process. Teleneurology can thus ensure timely access to specialist knowledge, particularly in supporting communication with families during this sensitive process. Future research should explore the integration of tele-neurointensive consultation into brain death diagnostics and its impact on organ donation processes, without replacing on-site neurointensive specialists.

Currently, there is no comprehensive reimbursement structure for teleneurocritical care in Germany. In our survey, only two networks reported receiving funding for their consultation services. The new G-BA center regulations may, for the first time, provide a sustainable reimbursement framework, although implementation and funding levels remain to be defined [17]. Rather than isolated, center-specific solutions, nationwide regulatory frameworks should be pursued by networks engaged in tele-ICU medicine. Interdisciplinary collaboration - a defining feature of these centers - offers neurology an opportunity to strengthen its role in intensive care and to contribute to regional care structures, similar to teleneurology in stroke care. As part of ongoing hospital reform with service-group designation across care levels, telemedical collaboration will likely gain importance. The experience from telestroke provides a solid foundation to extend these processes to interdisciplinary, cross-sector tele-ICU care.

This study has limitations. It is based on a survey conducted among established tele-stroke networks in Germany. However, not all of the 22 networks participated, which may limit the external validity of the findings. Furthermore, it is possible that networks not affiliated with the German Stroke Society were not reached. The results reflect the perspectives of network coordinators and may be subject to respondent and recall bias. Because no fixed recall window was specified, frequency reports may vary by respondents’ internal reference periods, limiting direct volume comparisons across networks.

## Conclusions

A promising avenue for the further development of teleneurocritical care lies in the targeted expansion of existing tele-stroke infrastructures. By adapting available technology and leveraging established organizational frameworks, new areas of application can be integrated. Key aspects such as neuroprognostication and the perspective of nursing care should be included in this process. A sustainable financing model is essential to ensure broad access to high-quality care for neurologically critically ill patients. The ongoing hospital reform in Germany may offer a timely opportunity to anchor this care model within future service structures.

## Supplementary Information

Below is the link to the electronic supplementary material.

Supplementary Material 1

Supplementary Material 2

Supplementary Material 3

Supplementary Material 4

Supplementary Material 5

## Data Availability

The datasets used and analyzed during the current study are available from the corresponding author on reasonable request.

## Abbreviations

CIP/CIM: Critical illness polyneuropathy/myopathy
COVID-19: Coronavirus Disease 2019
CT: Computed tomography
DGAI: The German Society of Anesthesiology and Intensive Care Medicine
DIVI: German Interdisciplinary Association for Intensive and Emergency Medicine
ERIC: Enhanced Recovery after Intensive Care
G-BA: Federal Joint Committee
ICU: Intensive care unit
MRI: Magnetic resonance imaging
SOS-TeleNET: Telemedicine stroke network East Saxony

## References

[1] Audebert, H. J., et al. (2005). Telemedicine for safe and extended use of thrombolysis in stroke: The telemedic pilot project for integrative stroke care (TEMPiS) in Bavaria. *Stroke*, *36*, 287–291. 15625294 10.1161/01.STR.0000153015.57892.66

[2] Barlinn, et al. (2021). Telemedizin in der Schlaganfallversorgung – versorgungsrelevant für Deutschland. *Der Nervenarzt*, *92*, 593–601. 34046722 10.1007/s00115-021-01137-6 PMC8184549

[3] Lilly, C. M., et al. (2011). Hospital mortality, length of stay, and preventable complications among critically ill patients before and after tele-ICU reengineering of critical care processes. *Journal of the American Medical Association*, *305*, 2175–2183. 21576622 10.1001/jama.2011.697

[4] Lilly, C. M. (2014). A multicenter study of ICU telemedicine reengineering of adult critical care. *Chest*, *145*, 500–507. 24306581 10.1378/chest.13-1973

[5] https://register.awmf.org/assets/guidelines/001-034l_S1_Telemedizin_in-der-Intensivmedizin_2021-01_1.pdf. Accessed: 24.02.2025.

[6] Schroeter, M., Klein, J., Erbguth, F., et al. (2021). Ergebnisse der 14. Erhebung der Deutschen gesellschaft für neurologie Zur struktur der neurologischen kliniken Mit Akutversorgungsauftrag in Deutschland. *DGNeurologie*, *4*, 332–344.

[7] Murray, N. M., et al. (2023). Teleneurocritical care for patients with large vessel occlusive ischemic stroke treated by thrombectomy. *Neurocritical Care*, *38*(3), 650–656. 36324004 10.1007/s12028-022-01632-x

[8] Murray, N. M., et al. (2024). Teleneurocritical care is associated with equivalent billable charges to in-person neurocritical care for patients with acute stroke. *Journal of Telemedicine and Telecare*, *30*(10), 1629–1635. 37032473 10.1177/1357633X231166160

[9] Pereira, A. J. (2024). Effect of Tele-ICU on clinical outcomes of critically ill patients: The TELESCOPE randomized clinical trial. *Journal of the American Medical Association*, *332*(21), 1798–1807. 39382244 10.1001/jama.2024.20651 PMC11581649

[10] https://www.dsg-info.de/ueber-uns/kommissionen/. Zugegriffen: 03.03.2025.

[11] Klingner, C. C., et al. (2020). Effektivität, effizienz und sicherheit der Schlaganfall-Telemedizin in Zeiten der Corona-Pandemie: Der „Fall Thüringen [Effectiveness, efficiency and safety of stroke telemedicine in times of the coronavirus pandemic: The case Thuringia]. *Der Nervenarzt*, *91*, 946–951. 32747988 10.1007/s00115-020-00970-5 PMC7397963

[12] Vollmuth, C., et al. (2021). Impact of the coronavirus disease 2019 pandemic on stroke teleconsultations in Germany in the first half of 2020. *European Journal of Neurology*, *28*, 3267–3278. 33619788 10.1111/ene.14787 PMC8013200

[13] Lang, S., et al. (2022). Thüringen: COVID-Konzept Senkt Mortalität. *Dtsch Arztebl*, *119*, A17–22.

[14] Pham, X., et al. (2022). Association of neurocritical care services with mortality and functional outcomes for adults with brain injury: A systematic review and Meta-analysis. *JAMA Neurol*, *79*, 1049–1058. 36036899 10.1001/jamaneurol.2022.2456 PMC9425286

[15] Adrion, C., et al. (2020). Enhanced recovery after intensive care (ERIC): Study protocol for a German stepped wedge cluster randomised controlled trial to evaluate the effectiveness of a critical care telehealth program on process quality and functional outcomes. *British Medical Journal Open*, *10*, e036096. 10.1136/bmjopen-2019-036096 PMC7520839 32978185

[16] https://innovationsfonds.g-ba.de/downloads/beschluss-dokumente/128/2022-01-21_ERIC_Ergebnisbericht.pdf. Zugegriffen: 24.02.2025.

[17] https://www.g-ba.de/downloads/39-261-6238/2023-10-19_Zentrums-Regelungen_Intensivmedizin-rechtsfoermliche-Aenderungen_BAnz.pdf. Zugegriffen: 24.02.2025.

[18] Eitenberger, M., et al. (2024). Focusing on experts: Expectations of healthcare professionals regarding the use of telemedicine in intensive care units. *Digit Health*, *10*, 20552076241257042. 38836049 10.1177/20552076241257042 PMC11149446

[19] Schulte, K., et al. (2018). Decline in organ donation in germany: A nationwide secondary analysis of all inpatient cases. *Deutsches Ärzteblatt International*, *115*, 463. 30064626 10.3238/arztebl.2018.0463 PMC6111206

[20] Darby, J. M., et al. (2021). Reliability of the telemedicine examination in the neurologic diagnosis of death. *Neurol Clin Pract*, *11*, 13–17. 33968467 10.1212/CPJ.0000000000000798 PMC8101299

[21] Matiello, M., et al. (2021). Teleneurology-enabled determination of death by neurologic criteria after cardiac arrest or severe neurologic injury. *Neurology*, *96*, e1999–e2005. 33637632 10.1212/WNL.0000000000011751

[22] Siminoff, L. A., et al. (2002). Families’ understanding of brain death. *Progress in Transplantation (Aliso Viejo, Calif.)*, *13*, 218–224. 10.1177/152692480301300309 14558637

