# Supplementary material for: Teleneurology expertise in intensive care units across Germany - a nationwide survey
Source: Neurol Res Pract. 2025 Nov 24;7(1):94. doi: 10.1186/s42466-025-00451-7 (PMC12645759; doi:10.1186/s42466-025-00451-7)
Supplement: Supplementary file 1 — Supplementary Material 1 [file 42466_2025_451_MOESM1_ESM.pdf]

„Tele-Neurointensiv-Konsil“:

Bedarfserhebung Netzwerkzentren

Antwort ID ____

Zentrum ____

Haben Sie eigenständige neurologische Intensivstation? □ ja □ nein

Wie viele Betten haben Sie auf Ihrer neurologischen Intensivstation? ____

Wie viele Beatmungsplätze davon sind auf Ihrer neurologischen Intensivstation? ____

Haben Sie eigenständige internistische/anästhesiologische Intensivstation? □ ja □ nein

Wie viele Betten haben Sie auf Ihrer internistischen/anästhesiologischen Intensivstation? □ <20, □ ≥20

Wie viele Beatmungsplätze sind davon auf Ihrer internistischen/anästhesiologischen Intensivstation? □ <20, □ ≥20

Haben Sie eine neurologische Konsiltätigkeit auf Ihrer internistischen/anästhesiologischen Intensivstation? □ ja □ nein

Hat der/die Neurolog:in eine Zusatzbezeichnung für Intensivmedizin? □ ja □ nein

Erhalten Sie Konsilanfragen von den Intensivstationen Ihrer Kooperationskliniken? □ ja □ nein

Wenn ja, dann: □ als regulären Termin, z.B. einmal pro Woche fixe Besprechung von Patienten, □ bei Bedarf, Regelarbeitszeit, □ bei Bedarf, 24/7 (=Notfallindikation), □ Indikationsgetriggert (Pflicht bei bestimmten Indikationen)

Zu welchen Fragestellungen werden Sie hinzugezogen? □ Schlaganfall, □ Hirnblutungen, □ Schädel-Hirn-Trauma, □ V.a. epileptisches Geschehen/Status epilepticus, □ Meningitis/Enzephalitis, □ CIP/CIM, □ Bewusstseinsstörung, □ Delir, □ protrahiertes Weaning, □ Beratung vor Therapiebeendigung, □ Beratung zur Prognoseabschätzung, □ Beratung zum Rehapotential, □ Mögliches Vorliegen eines irreversiblen Hirnfunktionsausfalls, □ Sonstiges

Ergebnis des Konsils: (bitte die prozentuale Häufigkeit im Kommentarfeld eintragen) Diagnostikempfehlung [Kommentar], Therapieempfehlung [Kommentar], Therapierückzug, [Kommentar], Folgekonsil vereinbaren [Kommentar], Übernahme [Kommentar]

Wer führt das Konsil durch? □ Oberarzt Neurologie, □ Facharzt Neurologie mit Zusatzbezeichnung Intensivmedizin, □ Facharzt Neurologie, □ Assistenzarzt Neurologie, □ Teleneurologe, der für Akutanfragen Schlaganfall zuständig ist, □ Transplantationsbeauftragte des Klinikums, □ anderer Facharzt, □ Sonstiges

Wie werden die Konsilanfragen bearbeitet? □ telefonisch, □ Telekonsil (Audio-Video-Kommunikation), □ vor Ort durch lokalen Konsilneurologen, □ vor Ort durch Neurologen aus dem Zentrum

Wenn Telekonsil, welche technische Umsetzung? □ Mitbeurteilung Bildgebung, □ Mitbeurteilung Tele-EEG, □ Sonstiges

Wurde die Kosillleistung finanziert? Wenn ja, dann: □ Konsilpauschale, □ Rahmenvertrag, □ Sonstiges

Erhalten Sie Konsilanfragen zur Abklärung eines möglichen irreversiblen Hirnfunktionsausfalls (IHA)? □ ja □ nein

Welche Maßnahmen leiten Sie im Rahmen eines möglichen IHA ein? □ telefonische Beratung mit Mitbeurteilung der Bildgebung, □ Telekonsil-Beratung (Audio-Video-Kommunikation), □ Beratung bzw. Untersuchung vor Ort, □ Kontakt zu Transplantationsbeauftragten, □ bei Bedarf Kontakt zu DSO, □ Sonstiges

Wären für Ihr Netzwerk eine Tele-Neurointensivkonsil interessant? □ ja □ nein

Zu welchen Fragestellung würden Sie gerne beraten? □ Bewusstseinsstörung, □ protrahiertes Weaning, □ Delir, □ V.a. epileptisches Geschehen/Status epilepticus, □ Beratung vor Therapiebeendigung, □ Schlaganfallpatienten, □ SHT-Patienten, □ Hirnblutungen, □ Beratung zur Prognoseabschätzung, □ Beratung zum Rehapotential, □ Mögliches Vorliegen eines irreversiblen Hirnfunktionsausfalls, □ CIP/CIM, □ Sonstiges

Wie viele Konsil würden schätzungsweise in Ihrem Netzwerk monatlich anfallen? _____________

Würden Sie das Pflegepersonal zur Beratung oder Schulung involvieren? □ ja □ nein

Wenn ja, dann zu welchen folgenden Neurointensiv-Pflegeaspekten? □ Dysphagiemanagement bei neurologischen Patienten, □ Management von Sedierung, Analgesie und Delir, □ Beobachtung und Scoring von neurologischen Krankheitsbildern, z.B. epileptische Anfälle, Ausfallsymptomatik, Schlaganfall, □ Sonstiges

Noch Hinweise/Vorschläge/Bedenken Ihrerseits: _____________________________
