# Supplementary material for: Teleneurology expertise in intensive care units across Germany - a nationwide survey
Source: Neurol Res Pract. 2025 Nov 24;7(1):94. doi: 10.1186/s42466-025-00451-7 (PMC12645759; doi:10.1186/s42466-025-00451-7)
Supplement: Supplementary file 2 — Supplementary Material 2 [file 42466_2025_451_MOESM2_ESM.pdf]

„Tele-Neurointensiv-Konsil“:

Bedarfserhebung Kooperationskliniken SOS-TeleNet

Antwort ID ____

Zentrum ____

Welche ITS? □ anästhesiologisch, □ internistisch

Wie viele Betten haben Sie auf Ihrer internistischen/anästhesiologischen Intensivstation?
□ <20, □ ≥ 20

Wie viele Beatmungsplätze sind davon auf Ihrer internistischen/anästhesiologischen Intensivstation? □ <20, □ ≥20

Haben Sie eine neurologische Konsiltätigkeit auf Ihrer internistischen/anästhesiologischen Intensivstation? □ ja, aus eigener Klinik, □ ja, aus externer Klinik, □ ja, niedergelassener Kollege, □ nein

Wenn ja, wie eingebunden? □ regulär, □ x-mal / Woche, □ auf Abruf

Zu welchen Themen? □ Bewusstseinsstörungen, □ Schlaganfall, □ SHT, □ Epilepsie, □ IHA, □ Delir, □ Weaning, □ Prognoseabschätzung, □ Therapiebeendigung, □ Rehapotenzial, □ Sonstiges

Wenn Sie das Angebot eines Tele-Neurointensiv-Konsils nutzen würden, dann gerne: □ regulärer Termin, □ nur bei Bedarf

Wieviel Konsile würden schätzungsweise anfallen ____

Das Pflegepersonal würde Beratung oder Schulung zu folgenden Neurointensivthemen wünschen:
□ Dysphagiemanagement, □ Delir, Analgesie und Sedierung, □ Monitoring Scoring

Zu welchen Themen besteht besonders Bedarf für Tele-Neurointensiv-Konsile?
□ Bewusstseinsstörungen, □ Schlaganfall, □ SHT, □ Epilepsie, □ IHA, □ Delir, □ Weaning, □ Prognoseabschätzung, □ Therapiebeendigung, □ Rehapotenzial, □ Sonstiges
