# Supplementary material for: Teleneurology expertise in intensive care units across Germany - a nationwide survey
Source: Neurol Res Pract. 2025 Nov 24;7(1):94. doi: 10.1186/s42466-025-00451-7 (PMC12645759; doi:10.1186/s42466-025-00451-7)
Supplement: Supplementary file 3 — Supplementary Material 3 [file 42466_2025_451_MOESM3_ESM.pdf]

Survey S1 (English) — "Tele-Neurocritical Consultation": Needs Assessment — SOS-TeleNET Spoke Hospitals

English translation of the original German questionnaire. Item order and response options preserved.

Response ID: ______________________________

Center: ___________________________________

Which ICU? ☐ anesthesiology ☐ internal medicine

How many beds does your ICU have? ☐ <20 ☐ ≥20

How many ventilator-capable beds does your ICU have? ☐ <20 ☐ ≥20

Do you have neurological consultation coverage on your ICU?

☐ yes, from our own hospital ☐ yes, from an external hospital ☐ yes, from a community neurologist ☐ no

If yes, how is it organized? ☐ regular (scheduled) ☐ X times per week ☐ on call/on demand

For which topics?

☐ disorders of consciousness ☐ stroke ☐ traumatic brain injury (TBI) ☐ epilepsy

☐ brain death (irreversible loss of brain function) ☐ delirium ☐ weaning

☐ prognosis estimation ☐ therapy limitation ☐ rehabilitation potential ☐ other: ___________

If you would use a teleneurocritical consultation service, would you prefer: ☐ regular scheduled appointments ☐ on demand only

How many consultations would you estimate? ____________________________

Nursing staff would like counseling/training on the following neuro-ICU topics:

☐ dysphagia management ☐ delirium, analgesia, and sedation ☐ monitoring and scoring

For which topics is there particular need for teleneurocritical consultations?

☐ disorders of consciousness ☐ stroke ☐ TBI ☐ epilepsy ☐ brain death (irreversible loss of brain function)

☐ delirium ☐ weaning ☐ prognosis estimation ☐ therapy limitation ☐ rehabilitation potential ☐ other: ___________
