# Supplementary material for: Teleneurology expertise in intensive care units across Germany - a nationwide survey
Source: Neurol Res Pract. 2025 Nov 24;7(1):94. doi: 10.1186/s42466-025-00451-7 (PMC12645759; doi:10.1186/s42466-025-00451-7)
Supplement: Supplementary file 4 — Supplementary Material 4 [file 42466_2025_451_MOESM4_ESM.pdf]

Supplementary Table S1. Structural characteristics of German telestroke networks (summary from Barlinn et al., 2021)

Source: Barlinn J et al., Telemedizin in der Schlaganfallversorgung – versorgungsrelevant für Deutschland, Der Nervenarzt 2021;92:593–601. This table extracts key structural, operational and financing details of German telestroke networks.

Domain	Item	Value (from Barlinn et al., 2021)
Scope & Coverage	Active telestroke networks (Germany)	22 networks
Scope & Coverage	Centers within networks	Total 43; per network median 1.5 (IQR 1–3)
Scope & Coverage	Cooperating hospitals	Total 225; per network median 9 (IQR 4–17)
Scope & Coverage	Hospital types	173 internal medicine; 52 neurology clinics
Scope & Coverage	Geographic access	Contribution to “near-home” access; maps provided in source (30-min reachability)
Operations	Availability	Teleconsultation available 24/7/365 in all networks
Operations	Minimum consultant qualification	Neurologist with board-certified standard (as minimum)
Operations	Primary indications	Suspected stroke in 21/22 networks (95.5%), often within therapeutic window
Operations	Time window focus	6/22 networks explicitly within ≤24h after symptom onset
Operations	Broader acute neurology	One network explicitly evaluates broader acute neurology beyond stroke

Operations	Transfer/transport models	Primarily Drip & Ship; additionally Mothership (3 networks) and Flying/Driving Interventional Team (2 networks)
Operations	Additional staffing at hubs	Median 2.5 FTE per network (IQR 1.5–4), including medical/nursing/therapy/admin
Activity	Teleconsultations (2018)	Total 38,211; per network median 1,340 (IQR 319–2,758)
Activity	Thrombolysis rate	14.1% (95% CI 13.6–14.7) among stroke patients
Activity	Transfers for thrombectomy	7.9% (95% CI 7.5–8.4) of ischemic stroke patients
Financing	Payer models (centers)	Regionally heterogeneous: per-case reimbursement in Saxony/Thuringia; add-on per stroke in Bavaria; 2 networks third-party funding; ~50% no dedicated reimbursement for centers (costs covered by partner hospitals)
Financing	Payer models (spoke hospitals)	OPS 8-98b ("complex stroke care with teleconsultation") reimburses additional workload at spoke hospitals in ~77% of networks; does not reimburse hub services
Quality & Governance	Quality assurance	All networks conduct QA; often annually; mix of mandatory (state-level) and voluntary/network-led QA; indicators aligned with ADSR
Quality & Governance	Public vs. for-profit status	Not explicitly reported in source
Quality & Governance	ICU vs. ER coverage	Source focuses on acute stroke pathways; ER emphasis; ICU-

specific coverage not systematically reported
