# Supplementary material for: Teleneurology expertise in intensive care units across Germany - a nationwide survey
Source: Neurol Res Pract. 2025 Nov 24;7(1):94. doi: 10.1186/s42466-025-00451-7 (PMC12645759; doi:10.1186/s42466-025-00451-7)
Supplement: Supplementary file 5 — Supplementary Material 5 [file 42466_2025_451_MOESM5_ESM.pdf]

Survey S1 (English) — “Tele-Neurocritical Consultation”: Needs Assessment — Network Centers

English translation of the original German questionnaire. Item order and response options preserved.

Response ID: ______________________________

Center: ___________________________________

Do you have a dedicated neurological intensive care unit (ICU)? ☐ yes ☐ no

How many beds does your neurological ICU have? ____________________________

How many of these are ventilator-capable beds? ____________________________

Do you have a dedicated internal medicine/anesthesiology ICU? ☐ yes ☐ no

How many beds does your internal medicine/anesthesiology ICU have? ☐ <20 ☐ ≥20

How many ventilator-capable beds are there on your internal medicine/anesthesiology ICU? ☐ <20 ☐ ≥20

Is there routine neurological consultation coverage on your internal medicine/anesthesiology ICU? ☐ yes ☐ no

Does the neurologist have an additional qualification in intensive care medicine? ☐ yes ☐ no

Do you receive consultation requests from ICUs at your cooperating hospitals? ☐ yes ☐ no

If yes, how are these scheduled/triggered?

☐ regular appointment (e.g., fixed weekly case review)

☐ on demand, during regular working hours

☐ on demand, 24/7 (emergency indication)

☐ indication-triggered (mandatory for defined indications)

For which questions/indications are you consulted?

☐ ischemic stroke ☐ intracerebral hemorrhage ☐ traumatic brain injury

☐ suspected epileptic events/status epilepticus ☐ meningitis/encephalitis ☐ CIP/CIM

☐ disorders of consciousness ☐ delirium ☐ prolonged weaning

☐ consultation before therapy limitation ☐ prognosis estimation ☐ rehabilitation potential

☐ possible brain death (irreversible loss of brain function) ☐ other: ___________

Outcome of the consultation (please indicate approximate percentage frequency in the comment field):

Diagnostic recommendation [comment: ______________________________]

Treatment recommendation [comment: ______________________________]

Limitation/withdrawal of therapy [comment: __________________________]

Follow-up consultation scheduled [comment: _________________________]

Patient transfer/assumption of care [comment: ________________________]

Who conducts the consultation?

☐ senior/attending neurologist ☐ board-certified neurologist with additional intensive care qualification

☐ board-certified neurologist ☐ neurology resident

☐ teleneurologist primarily responsible for acute stroke requests

☐ hospital transplant coordinator ☐ other specialist ☐ other: ___________

How are the consultation requests handled?

☐ by telephone ☐ teleconsultation (two-way audio-video) ☐ on site by local consulting neurologist

☐ on site by neurologist from the hub/center

If teleconsultation is used, which technical components?

☐ remote imaging review ☐ remote EEG review (tele-EEG) ☐ other: ___________

Is the consultation service reimbursed/financed? If yes:

☐ per-consultation flat rate ☐ framework agreement ☐ other: ___________

Do you receive consultation requests regarding the assessment of possible brain death (irreversible loss of brain function)? ☐ yes ☐ no

Which measures do you initiate when brain death is suspected?

☐ telephone advice with remote imaging review ☐ teleconsultation (audio-video communication)

☐ on-site consultation/examination ☐ contact with the hospital transplant coordinator

☐ contact with the German Organ Transplantation Foundation (DSO) as needed ☐ other: ___________

Would a teleneurocritical consultation service be of interest for your network? ☐ yes ☐ no

Which topics would you like to receive consultation on?

☐ disorders of consciousness ☐ prolonged weaning ☐ delirium

☐ suspected epileptic events/status epilepticus ☐ consultation before therapy limitation

☐ stroke patients ☐ TBI patients ☐ intracerebral hemorrhage

☐ prognosis estimation ☐ rehabilitation potential ☐ possible brain death (irreversible loss of brain function)

☐ CIP/CIM ☐ other: ___________

How many consultations would you estimate per month in your network? ___________

Would you involve nursing staff for counseling or training? ☐ yes ☐ no

If yes, which neuro-ICU nursing topics?

☐ dysphagia management in neurological patients

☐ management of sedation, analgesia, and delirium

☐ observation and scoring of neurological conditions (e.g., seizures, focal deficits, stroke)

☐ other: ___________

Additional notes/suggestions/concerns: ____
